# Oral Health Statuses of Children and Young Adults with Autism Spectrum Disorder: An Umbrella Review

**DOI:** 10.3390/jcm13010059

**Published:** 2023-12-22

**Authors:** Waqas Sami, Mohammad Shakil Ahmad, Riyaz Ahamed Shaik, Mohammad Miraj, Sadiya Ahmad, Muhammed Hamza Molla

**Affiliations:** 1 Department of Pre-Clinical Affairs, College of Nursing, QU-Health, Qatar University, Doha P.O. Box 2713, Qatar; waqas@qu.edu.qa; 2 Department of Family and Community Medicine, College of Medicine, Majmaah University, Majmaah 11952, Saudi Arabia; m.shakil@mu.edu.sa (M.S.A.); r.shaik@mu.edu.sa (R.A.S.); 3 Department of Physical Therapy and Health Rehabilitation, College of Applied Medical Sciences, Majmaah University, Majmaah 11952, Saudi Arabia; m.molla@mu.edu.sa; 4 Department of Psychology, University of Windsor, Windsor, ON N9B 3P4, Canada; ahmad81@uwindsor.ca; 5 College of Dental Medicine, QU-Health, Qatar University, Doha P.O. Box 2713, Qatar

**Keywords:** autism spectrum disorder, oral health, dental caries, periodontal disease, umbrella review, prevalence, integrated care, dental treatment, policy formulation

## Abstract

This study aimed to comprehensively evaluate the oral health statuses of children and adults within the autism spectrum disorder (ASD) population through an umbrella review approach. The prevalence of dental caries, periodontal disease, and associated variables were investigated across selected studies. A systematic search was conducted across databases including PubMed, Scopus, EMBASE, Science Citation Index, Science Direct, Web of Science, MEDLINE, and Wiley Online Library to identify relevant studies. The assessed variables included dental caries prevalence, periodontal disease prevalence, oral hygiene indicators, and the necessity of dental treatment. The pooled prevalence rates, odds ratios, and standardized mean differences were calculated where applicable. The pooled prevalence of dental caries among ASD individuals ranged from 60.6% to 67.3%, while the periodontal disease prevalence ranged from 59.8% to 69.4%. High rates of dental treatment under general anesthesia were reported. Heterogeneous dental caries and periodontal disease prevalence rates were identified, highlighting the need for collaboration and preventive care. Several studies also reported higher prevalence rates of dental trauma and self-inflicted oral injuries among individuals with ASD. However, the review also identified significant methodological limitations in the included studies, including inconsistency in oral health assessment methods and potential bias. The necessity for targeted policies due to high prevalence rates and the requirement for integrated care systems in high DMFT regions were also observed. The umbrella review synthesized diverse findings, revealing variations in dental caries and periodontal disease prevalence among ASD individuals. This review underscores the need for tailored interventions and policies to address oral health disparities. It highlights the necessity of integrated care systems, methodological improvements, and longitudinal studies to comprehensively address the multifaceted oral health challenges within the ASD population.

## 1. Introduction

The oral health conditions of individuals with autism spectrum disorder (ASD) have become a serious issue that requires in-depth research [1]. A wide variety of neurodevelopmental diseases, including autism spectrum disorder, are characterized by difficulties in communicating, limitations in behavior, and impairments in social interaction [1]. The relationship between ASD and dental health is complex, and it may be impacted by things like sensory sensitivity, communication difficulties, and behavioral problems [2]. Although various studies have examined the oral health outcomes of people with ASD, a synthesis of the available information is still necessary to guide the development of specific interventions and policies [3].

Self-harming behaviors, anger, and tantrums are among the severe behavioral problems that are frequently associated with ASD and are triggered by everyday environmental signals, increased arousal, or stressors [4]. As a result of the severity of the disorder, more than 60% of children with ASD engage in self-harming behaviors, such as severe self-biting, self-pinching, and scratching [5,6]. Bruxism, tongue thrusting, gingival manipulation, and lip biting are all common detrimental oral habits [7], which increase a person’s susceptibility to periodontal disease, tooth trauma, and other oral illnesses.

Notably, the difficulties faced by people with ASD in receiving dental care are exacerbated by the complex interaction of behavioral issues, co-occurring disabilities, and financial considerations [8,9]. Exaggerated unpleasant responses to visual, aural, and tactile inputs during dental interactions are caused by anomalies in sensory processing [8,9]. Nevertheless, many people on the ASD spectrum can successfully receive dental therapies in common clinical environments by using a variety of behavioral management tactics. In order to modify dental procedures for children with ASD diagnoses, it is essential to use basic behavioral approaches like tell–show–do, rewarding behavior, and desensitization [9,10,11]. Visual pedagogy, social narratives, and applied behavioral analysis are specialized therapeutic pedagogical approaches that have shown promising results in facilitating dental assessments and basic preventative treatments for the ASD population [2,4,11].

The main goal of this umbrella review is to thoroughly compile and evaluate the existing literature in order to shed light on the oral health conditions of children and young people with ASD. We intend to distil and synthesize information from numerous individual studies using a thorough umbrella review methodology in order to identify the patterns, trends, and discrepancies across various oral health markers.

## 2. Materials and Methods

### 2.1. Review Protocol

**Figure 1:**
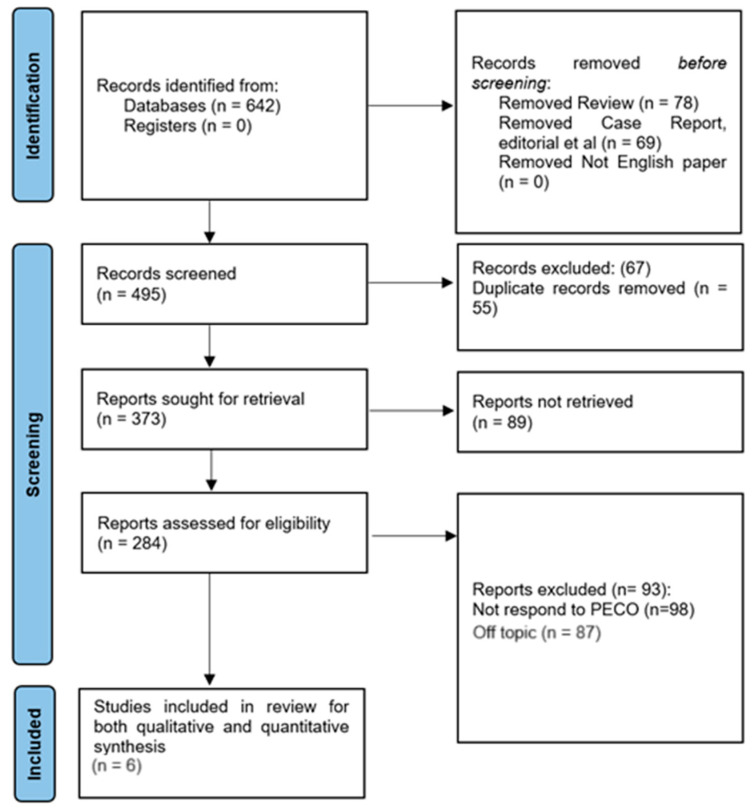
PRISMA protocol representation for this umbrella review.

This umbrella review adheres to the Preferred Reporting Items for Systematic Reviews and Meta-Analyses (PRISMA) protocol [12]. This review process follows a systematic and comprehensive methodology, guided by the PRISMA framework, as depicted in Figure 1. The impending registration of this review with PROSPERO is currently in progress, as the necessary documentation has been submitted for registration with the following registration number: 467566.

### 2.2. PICOS Strategy

The PECO (Population, Exposure, Comparator, Outcome) protocol for this study included a thorough examination of the state of oral health among people in the pediatric and young adult age cohorts who suffer from ASD. People with ASD from a wide age range, including children and young adults, made up the group under investigation. The topic of study included the many ASD correlations and manifestations, taking into account factors like behavioral characteristics, sensory sensitivities, pharmacological therapies, and related comorbidities. This review aims to compare the oral health status of people on the ASD spectrum to that of neurotypical peers or people who are not impacted by ASD. The outcome measures included, but were not limited to, dental caries prevalence and severity, periodontal status, oral hygiene indices, oral pathology prevalence, treatment modalities, and the influence of ASD-specific behaviors on oral health outcomes. In order to give a thorough review of the interactions between ASD and oral health parameters in children and young adults, the we tried to encompass the various facets of oral health status in the synthesized body of information.

### 2.3. Database Search Strategy

Eight electronic databases were searched using advanced search techniques and Boolean operators to locate relevant content. MeSH (Medical Subject Headings) keywords were carefully chosen to enhance search relevancy and accuracy. Systematically searching PubMed, Scopus, EMBASE, Science Citation Index, Science Direct, Web of Science, MEDLINE, and Wiley Online Library was the first step in the search. To combine pertinent terms and guarantee thorough coverage, Boolean operators like “AND” and “OR” were wisely used. The most common search terms were various forms of “autism spectrum disorders”, “oral health”, and “children and young adults”. MeSH phrases were used to make it easier to include controlled vocabulary, improving search specificity and the strategy. Search terms including “autism spectrum disorders” OR “autism” OR “ASD” AND “oral health” OR “dental health” OR “oral hygiene” AND “children” OR “adolescents” OR “young adults” comprised the entire search strategy. The retrieval of research addressing oral health indicators, dental caries prevalence, periodontal disease, and related variables within the ASD community was made easier via the combined MeSH and keyword search. This approach made sure that a thorough analysis of the relevant literature was conducted across numerous databases, enabling a thorough and complex synthesis of the available data in the umbrella review.

### 2.4. Selection Criterion for the Review

To guarantee that studies that matched the review’s objectives and scope were chosen, inclusion and exclusion criteria for this investigation were systematically defined. To ensure methodological rigor and transparency in the study selection, these criteria were crucial.

Inclusion criteria:

- Population: Research focusing on individuals with autism spectrum disorder (ASD) and in children and young adults was eligible for inclusion.
- Study Focus: Selected papers were required to evaluate the prevalence rates of dental caries, periodontal disease, and related oral hygiene indicators in people with autism spectrum disorder (ASD).
- Peer-reviewed research articles that have been published in scholarly publications were added, encouraging the utilization of reliable and verified research.
- Cross-sectional, cohort, case–control, and clinical trial study types were all taken into consideration, as well as observational and interventional study designs.

Exclusion criteria:

- Irrelevant Population: Research on children and young adults with ASD was expressly omitted from the study.
- Relevant Outcome: Studies were excluded if they did not evaluate the prevalence rates of dental caries, periodontal disease, and related oral hygiene indicators, among other oral health indicators.
- Publication Type: In order to preserve the integrity of the research, non-peer-reviewed materials such as conference abstracts, editorials, and letters were disregarded.
- Non-English Language: Studies that were published in tongues other than English were disregarded since there might be difficulties in understanding and interpreting the results owing to language issues.
- Studies involving animals were not included because they did not directly address the oral health status of the ASD community, such as animal models or in vitro trials.

### 2.5. Data Extraction and Reviewer Protocol

To ensure consistency, accuracy, and rigor in the gathering of pertinent data from the chosen studies, the data extraction protocol used for this umbrella review was systematically designed. In the population of people with ASD, this protocol sought to record important variables and results related to oral health indicators and the prevalence rates of dental caries, periodontal disease, and related oral hygiene parameters. In order to extract pertinent data from each chosen study, a standardized data extraction form was created. The factors included the study’s author and year of publication, the study’s design, participant demographics (age range, sample size), the particular oral health indicators evaluated, the measurement tools used, the prevalence rates of dental caries and periodontal disease, the oral hygiene parameters, and whether or not general anesthesia was required for any dental work. To increase dependability and reduce bias, the data extraction process was carried out by two impartial reviewers. Data extraction was cross-verified for precision and comprehensiveness. The integrity of the extracted data was ensured by reaching an agreement through conversations among the reviewers in cases where there were differences. Interrater Reliability Test: A subset of studies (*n* = 10) was randomly chosen in order to evaluate the interrater reliability of the data extraction procedure. Data from these studies were separately extracted by the reviewers, and the outcomes were compared. The level of agreement between the reviewers was measured using Cohen’s Kappa coefficient. The coefficient showed a high degree of agreement (=0.75), confirming the accuracy and consistency of the data extraction procedure. The interrater reliability test was used to check that the data extraction technique produced reliable and consistent results across the chosen studies. The review’s methodological transparency and scientific rigor were upheld via the careful implementation of this methodology, which also improved the reliability and validity of the findings that were synthesized.

### 2.6. Bias Assessment of Included Studies

**Figure 2:**
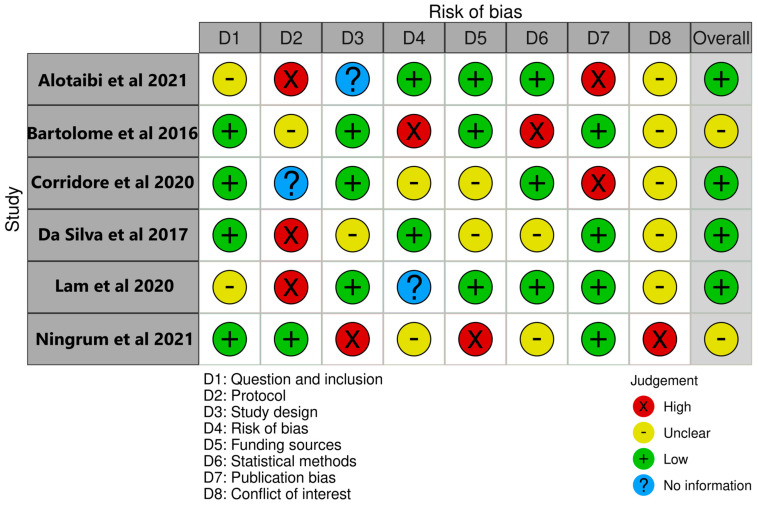
Bias assessment of the selected reviews [14,15,16,17,18,19].

The bias assessment process for this study was followed as per the Joanna Briggs Institute’s (JBI) Critical Appraisal Checklist for Systematic Reviews and Research Syntheses [13]. To increase the review’s robustness and reliability, this methodology was carefully carried out to assess the methodological qualities, bias risks, and potential limits of the chosen studies. Each chosen study went through a thorough bias evaluation procedure based on the JBI criteria, the details of which are furnished in Figure 2. The studies were critically evaluated by two independent reviewers who looked at important factors such as the study design, sampling techniques, measurement tools, statistical analysis, and potential sources of bias. For each criterion, the reviewers independently assigned scores and noted their findings.

## 3. Results

A total of 642 documents were initially found across multiple databases. Register records, however, were not discovered. Among the detected records, 69 were discarded because they were either case reports, editorials, or other works of the same kind, and 78 were eliminated because they were classified as reviews. A total of 495 records underwent additional evaluation after being found relevant following the initial screening. A total of 373 unique reports were left after 55 duplicate records were eliminated. Sadly, only 284 of these reports were evaluated for eligibility because 89 of them could not be satisfactorily recovered. A total of 93 reports were disregarded during the review based on predetermined standards. Among them, 89 reports were deemed off-topic for this evaluation, and 98 papers failed to address the PECO framework. Finally, six articles [14,15,16,17,18,19] were chosen for the study evaluation that comprised both a qualitative and quantitative synthesis. These studies supplied data that were pertinent and appropriate for the synthesis and met all of the requirements.

A summary of the chosen studies and their main conclusions is presented in Table 1. Each study’s reference number was used to evaluate several aspects of oral health among people with ASD. The table lists the precise factors evaluated, the data sources used, the main findings of each study, and the inferences made in light of the findings. This table provides a comprehensive understanding of the dental health issues that people with ASD must contend with. It efficiently summarizes the various facets of oral health that were examined during the studies. On the other hand, Table 2 provides a unified analysis of the data from the chosen studies, highlighting certain facets of oral health such as dental caries, periodontal disease, and oral hygiene.

### 3.1. Demographic Characteristics

The assessed variables under the selected papers provided a comprehensive assessment of oral health, including hygiene, dental caries, periodontal disease, malocclusion, oral habits, dental trauma, and the necessity of treatment [14,15,16,17,18,19]. The gingival and plaque indices were assessed [14], providing insight into the overall oral hygiene and presence of bacterial biofilms, which can contribute to caries and periodontal disease. Multiple studies evaluated the prevalence of dental caries [15,16,17,19], which is a common oral health issue, in children and individuals with ASD. This offered an understanding of the dental decay situation in the ASD population. Periodontal disease was another area of focus [15,16,17,19], further extending the scope of assessment into gum health and potential tooth loss.

The review also evaluated the prevalence of using general anesthesia [16], an indicator of the severity of dental procedures required, and often an indicator of more complex or advanced oral health issues. The prevalence of bruxism [17], which can contribute to tooth wear, was also evaluated. The other variables that were assessed included the salivary pH levels [17], which can influence the oral microbiome and caries development, and dental trauma prevalence [17], reflecting potential behavioral or coordination issues in the ASD population. The salivary flow rate and buffering capacity [17] were also evaluated, which could affect oral moisture and the ability to neutralize acids, respectively. Malocclusion prevalence [15,19], caries severity [15,19], oral cleanliness [19], periodontal condition [19], prevalence of gingival bleeding [19], and the decayed, missing, and filled permanent teeth (DMFT) index [19] were also examined, providing a broad overview of the oral health statuses in individuals with ASD.

### 3.2. Parametrical Assessment of Oral Health Indices

AlOtaibi et al. [14] did not clearly specify findings related to dental caries. For periodontal disease, this study found significantly higher gingival index values in children and adolescents with ASD compared to those without ASD, although the pooled standardized mean difference (SMD) indicated no significant difference in the mean values of the gingival index between the groups (SMD = −0.528, t = 1.381, *p* = 0.168). The oral hygiene assessment showed significantly higher plaque index values in the ASD group, but again, the pooled SMD showed no significant difference between the ASD and control groups (SMD = −0.687, t = 1.518, *p* = 0.129). Bartolomé et al. [15] found discrepancies in the studies regarding dental caries in children with ASD, with some studies indicating a higher incidence in the ASD group and others showing no significant difference or even a greater predisposition in the control group. For periodontal disease, the study found significantly worse periodontal statuses in children with ASD, especially those with sensory impairments. Oral hygiene was generally worse in the ASD group, although one study found no significant difference between the groups.

Corridore et al. [16] reported no common decayed, missing, and filled teeth (DMFT) or decayed, missing, and filled primary teeth (dmft) for the ASD group, with values varying by age relative to the control group. The periodontal disease assessment showed higher plaque index (PI) and gingival index (GI) values for the ASD group, indicating a greater incidence of periodontal disease. Oral hygiene was generally poor in the ASD group, with a high prevalence of gingivitis and high Simplified Oral Hygiene Index (OHI-S) scores. Da Silva et al. [17] reported a pooled prevalence of dental caries of 60.6% (95% CI: 44.0–75.1), which increased to 67.3% after the sensitivity analysis. The pooled prevalence of periodontal disease was 69.4% (95% CI: 47.6–85.0), which decreased to 59.8% after the sensitivity analysis. No significant difference was observed in oral hygiene status between the ASD and control groups.

Lam et al. [18] found no statistical difference in dental caries between the ASD and control groups. The study found a higher value for the Simplified Oral Hygiene Index of Treatment Needs (CPITN) in the ASD group (OR = 5.72), indicating worse periodontal health. Despite the poor oral hygiene in the ASD group, there was no significant difference in the OHI-S (OR = 4.04) or plaque index (WMD = 0.31) compared to the control group. Ningrum et al. [19] reported a higher DMFT index in the special needs group than in the control group (SMD = 0.441), indicating worse caries status. Periodontal health was also worse in the special needs group, with a higher CPITN (SMD = 1.419). The study found higher OHI-S scores (SMD = 0.803) and a higher plaque index (SMD = 0.158) in the special needs group, indicating poorer oral hygiene, while the gingival index showed evidence of publication bias.

## 4. Discussion

This review’s assessments provided important new information about the oral health statuses of people with ASD. The analysis highlights the complex issues involved in managing oral health within the ASD population by highlighting the variations in the frequency of dental caries and periodontal disease as well as the high rates of dental treatment under general anesthesia. These findings highlight the urgent need for customized methods of providing oral healthcare, calling for procedures that take into account the special requirements and behavioral traits of people with ASD. The consequences of the umbrella review also include intervention and policy-making techniques. The need for specialized oral health strategies, interventions, and preventive measures is highlighted by the frequency of dental caries and periodontal disease among people with ASD. Understanding the need for integrated and equitable care systems to preserve oral health, especially in areas with a high prevalence of caries, highlights the necessity of cooperative efforts among healthcare providers, educators, carers, and policymakers. By thoroughly synthesizing the available research, this overarching review provides a solid framework for the creation of evidence-based policies and interventions catered to the unique oral health needs of the ASD population.

It is also important to acknowledge the umbrella review’s contribution to the scientific conversation. This review encourages researchers to adopt improved designs, procedures, and reporting standards by emphasizing the limits of the available data, such as study heterogeneity, bias risks, and the need for more rigorous research methodologies. In order to fully understand the many facets of oral health within the setting of ASD, it is imperative that longitudinal research, standardized assessment techniques, and coordinated interdisciplinary investigations are conducted.

AlOtaibi et al. [14] found significant heterogeneity among the studies they investigated. They reported no significant differences in the mean values of both the gingival index (GI) and plaque index (PI) between the ASD and control groups, contradicting the notion of worse oral health statuses in individuals with ASD. Yet, the authors concluded that individuals with ASD need better access to oral healthcare and that further investigations are needed to understand how ASD affects gingival health and caries risk. Bartolomé et al. [15] identified discrepancies across studies regarding dental caries in children with ASD. Ten of the studies they reviewed reported worse states of oral hygiene in children with disabilities. They also noted a higher incidence of dental trauma in children with visual impairment and a greater frequency of self-inflicted injuries in soft tissues in autistic patients. Despite these findings, the authors remarked on the need for a larger number of research studies to corroborate these results due to the discrepancies found.

Several aspects of oral health, such as the prevalence rates of dental caries and periodontal disease, the need for treatment, and the frequency of general anesthesia use, were evaluated by Corridore et al. [16] in children with ASD. Their results showed that children with ASD had a heterogeneous distribution of decayed, filled, and missing teeth (DFMT) and dmft scores. Children with ASD also showed higher Periodontal Indexes (PI and GI), suggesting a higher frequency of periodontal disease. The study also showed a significant incidence of general anesthesia as a result of collaboration issues. In order to improve the oral health conditions of ASD patients, the authors stressed the need for improved teamwork, preventive care, and the creation of protocols specifically targeted to these patients. Assessing the frequency of dental caries and periodontal disease in people with ASD was the main focus of Da Silva et al.’s study [17]. Their data showed that there was a high frequency of periodontal disease (69.4%) and dental caries (60.6%) among people with ASD. The results of the sensitivity analysis revealed this predominance even more. In order to explicitly address the high prevalence of dental caries and periodontal disease in this group, the authors argued for the creation of an oral health policy. Lam et al. [18] investigated a wide range of oral health factors in people with ASD, including bruxism, salivary pH levels, the prevalence of tooth damage, and others. Their results showed that bruxism was more common and salivary pH levels were lower in ASD youngsters than in the controls. However, few other evaluated variables showed any evidence of substantial differences. The study emphasized the need for more research to fully comprehend the oral health statuses of people with ASD and emphasized the scarcity of data and the significant risk of bias. The DMFT index in special needs children with intellectual disabilities or ASD was the topic of Ningrum et al.’s study [19]. Their study found a link between the frequency of caries and the average DMFT value in each of their individual countries, highlighting the significance of an integrated and equitable treatment system to maintain oral health among children with special needs, especially in high DMFT countries.

A confluence of complex causes leads to a greater frequency of periodontal disease and tooth caries in the ASD community. This convergence includes eating habits that lean towards cariogenic substances and difficulties in maintaining oral hygiene, which are regularly experienced in the world of people with special needs [20]. The thorough examination of the studies included in this review and its analysis as a whole highlight the universal issue of the investigated people’s poor oral hygiene practices. The obvious existence of visible plaque at the point of data collection supports this notion. The observed increased plaque index may be explained by difficulties resulting from impaired manual dexterity or by both the individuals and their parents’ or carers’ disregard for recommended dental hygiene procedures [21].

Oral hygiene practices are significantly less effective when oral aversion or sensory hypersensitivity are present close to the oral cavity [22,23,24]. This compromises oral health. These difficulties are accentuated by reports showing a lack of interest in medication use and oral hygiene practices among this cohort [25]. The effects of such trends include decreased salivary flow, which may lead to an increased susceptibility to oral diseases, including dental caries [26]. Notably, the hesitation to seek dental care among these people raises an important problem. This hesitation results from the lack of interest in oral hygiene displayed by people with ASD and those who care for them. This hesitation leads to a high frequency of unmet dental needs among this cohort, along with potential financial concerns and impediments such as geographic distance and transportation [22,27].

Many people with ASD frequently show noticeable difficulties in daily living that exceed their cognitive capacities [28]. The developmental paradigm for autism exhibits significant individual variation in the impairment of executive functions [29]. The possibility of therapeutic intervention through occupational therapy throughout early life emerges as a feasible path within such situations. This strategy has the potential to improve social integration, ease difficulties with career pursuits, and lessen the chance of running into psychiatric comorbidities [30].

A sizeable fraction of affected children shows a family history of allergies, food intolerances, and autoimmune diseases such as psoriasis, rheumatoid arthritis, celiac disease, and Takayasu’s arteritis. These findings highlight the possibility that the immune system is involved in the etiopathogenesis of autism [31]. Furthermore, it is hypothesized that children and teenagers with ASD have heightened sensitivities to oral diseases. This vulnerability is brought on by greater barriers to receiving dental care, which has a negative impact on oral health outcomes [32].

Malocclusion and the associated orthodontic implications in the case of ASD individuals were some issues that we could not touch upon in our review; however, several other reviews have discussed it at length [33,34,35]. Barros et al. [33] and Da Motta et al. [34] focused specifically on the prevalence of malocclusion in individuals with ASD. Like in our review, Barros et al. [33] identified significant heterogeneity among the studies, which made it challenging to conclusively establish a risk of malocclusion in individuals with ASD. This finding aligns with the heterogeneity and discrepancies in the oral health parameters observed in our review, reflecting the inherent complexity in studying oral health in ASD populations.

Da Motta et al. [34], on the other hand, identified specific malocclusion characteristics that were more prevalent in individuals with ASD, including crowding and increased maxillary overjet. They also found that individuals with ASD had higher odds of Angle’s Class II and III malocclusion, open bite, and increased maxillary overjet than individuals without ASD. These findings suggest a potential link between ASD and certain malocclusion characteristics, a topic that our review did not specifically address. This discrepancy highlights the need for a more comprehensive approach to understand oral health in ASD, including not only general oral health parameters but also specific conditions such as malocclusion.

Erwin et al. [35] focused on the factors influencing oral health (OH) behaviors and the access to and delivery of dental care for children and young people with ASD. They identified several themes, including affordability and accessibility, autism-related factors, the dental environment, and the attitudes and knowledge of dental health professionals. These themes resonate with the findings of our review, which highlight the need for more inclusive and accessible oral healthcare services for individuals with ASD, as well as the importance of specialized training for dental professionals. However, Erwin et al.’s study [35] also emphasized the roles of cognitive or motor skill differences and sensory sensitivities in ASD, which our review did not specifically address. This underscores the need for future research to take into account these factors when studying oral health in ASD populations.

This umbrella review is not without its limitations, which call for careful attention in the interpretation and application of its findings. The intrinsic variety among the chosen studies in terms of the methodologies, sample sizes, and diagnostic criteria used is one major drawback. The reported prevalence rates and outcomes are rather variable as a result of this heterogeneity, which may have an impact on how broadly applicable the synthesized findings are. The conclusions of the review may also be impacted by publication bias, which occurs when studies with statistically significant results are more likely to be published. This bias may cause an overestimation of the observed effect sizes or prevalence rates, which would then affect the validity of the combined results and any recommendations that follow. Furthermore, because the umbrella review heavily relies on the data and methodology of the main studies that are included, any flaws or restrictions in those studies automatically affect the review as a whole. The quality assessment and risk of bias assessment across the chosen papers are common limitations of the umbrella review. The caliber and rigor of the original studies that are included in the review will determine the strength of the conclusions it may draw. It is difficult to determine a causal link between ASD and certain outcomes in oral health because of variations in the study designs, control groups, measurement techniques, and potential confounding factors. The included studies may not have fully taken into consideration the temporal and contextual aspects driving oral health inequalities among people with ASD, which could have limited the review’s insights. Furthermore, variations in the case definitions and sample makeup may be introduced because the chosen studies do not all use a single assessment tool or a set of standardized diagnostic criteria for ASD. Direct comparisons and drawing broad inferences are made more difficult by this heterogeneity. It is important to note that the review’s scope is limited to the information and results provided in the included research, potentially omitting subtle aspects that might have substantial impacts on the disparities in oral health among people with ASD.

Few important recommendations can be ascertained as per the findings of this umbrella review, which was also one of our secondary aims through this investigation. It underscores the need for more rigorous and standardized research methodologies to overcome the observed discrepancies and heterogeneity among different studies. Thus, one of the primary recommendations for future research is the development and utilization of standardized assessment criteria. This could ensure more consistency in measuring and reporting oral health parameters, such as the gingival index, plaque index, and decayed, missing, and filled teeth. This review also reveals high incidences of dental caries and periodontal disease in individuals with ASD, suggesting the need for effective preventive strategies. Future research should thus focus on identifying risk factors and developing tailored preventive interventions. It would also be valuable to investigate the impact of specialized training for dental professionals on improving oral health outcomes for individuals with ASD. Additionally, this review highlights the need for more inclusive and accessible oral healthcare services. Future research should explore strategies to enhance access to dental care for individuals with ASD, including the design of more accommodating dental care environments and the development of protocols for managing dental procedures under general anesthesia. The association between ASD and certain oral health conditions, such as a higher bruxism prevalence and lower salivary pH levels, suggests a need for further exploration of the physiological and behavioral aspects of ASD that may contribute to these conditions. This could lead to more effective management strategies and therapies. Ultimately, this investigation indicates that special needs children, especially those with ASD or intellectual disabilities, have higher dental caries prevalence rates. This finding calls for an integrated and equitable oral healthcare system, particularly in countries with high caries prevalence rates. Future studies should therefore investigate the effectiveness of such integrated care systems in improving oral health outcomes for this vulnerable population.

## 5. Conclusions

Conclusively speaking, this investigation encompasses a wide range of findings that collectively improve our comprehension of the complex relationship between oral health and ASD. This review offers essential insights into the complex difficulties faced by members of the ASD population with regard to their oral health through the rigorous analysis of a few chosen studies. The combined data emphasize the variations in dental caries and periodontal disease prevalence among people with ASD. This variation demonstrates the nuanced interactions among variables including age, behavioral traits, and dental hygiene practices in this population. The increased frequency of dental procedures performed under general anesthesia emphasizes the special needs of managing oral health in people with ASD, demanding customized strategies that meet the unique problems given by behavioral and sensory sensitivities. This review’s findings highlight the urgent need for focused interventions that are guided by evidence-based regulations in order to address the greater prevalence rates of dental caries and periodontal disease seen in people with ASD. The need for comprehensive healthcare strategies and interdisciplinary teamwork to reduce oral health inequities within this group is underlined by the demand for integrated and equitable care systems, particularly in areas with high caries prevalence rates.

## Figures and Tables

**Table 1:** Overview of the selected papers and their assessments.

Study	Variables Assessed	Data Sources	Key Findings	Conclusion
AlOtaibi et al. [14]	Gingival and plaque indices	MEDLINE, PubMed, the Cochrane Library, Web of Science, Controlled-Trial Database, Clinical Trials-US National Institutes of Health, National Institute for Health and Clinical Excellence, Virtual Health Library, and Saudi Digital Library	- Significant heterogeneity among the five studies. - No significant difference in the mean values of gingival index (GI) between the ASD and control groups - No significant difference in the mean values of plaque index (PI) between the ASD and control groups.	Individuals with ASD need help and better access to oral healthcare. Further investigation is needed with regard to gingival health in individuals with ASD and caries risk assessment to understand how this disorder affects oral health.
Bartolomé et al. [15]	Oral hygiene, dental caries, malocclusion, oral habits, dental trauma, and gingival/periodontal status	PubMed/Medline, Scopus, Cochrane Library	- Only the states of oral, gingival, and/or periodontal hygiene were considered worse in patients with ASD - Discrepancies found in studies regarding dental caries in children with ASD - Ten studies examined the oral hygiene index, and almost all found a worse state of oral hygiene in children with disabilities analyzed - Differences in the evaluation of the gingival statuses of children with ASD and the control group - Higher incidence of dental trauma in children with visual impairment - Greater frequency of self-inflicted injury in soft tissues and self-injurious habits in autistic patients	A larger number of research studies is needed to corroborate these results. There are discrepancies about the results found in the different studies regarding dental caries and oral hygiene in children with ASD and SD.
Corridore et al. [16]	- Prevalence of dental caries in children with ASD - Prevalence of periodontal disease in children with ASD - Necessity of treatment - Prevalence of using general anesthesia	Pubmed, Scopus, EMBASE, Science Citation Index, Science Direct, Web of Science	- Heterogeneous DFMT and dmft in ASD children - Higher Periodontal Indexes (PI and GI) in ASD children - High incidence of general anesthesia due to lack of collaboration	Lack of protocols for ASD patients, need for improved collaboration and preventive care.
Da Silva et al. [17]	- Prevalence of dental caries in individuals with ASD - Prevalence of periodontal disease in individuals with ASD	MEDLINE/PubMed, Web of Science, Scopus	- Dental caries prevalence: 60.6% - Periodontal disease prevalence: 69.4%	Need for oral health policies targeting high prevalence of dental caries and periodontal disease in ASD individuals.
Lam et al. [18]	- Prevalence of bruxism - Salivary pH levels - Dental trauma prevalence - Salivary flow rate - Buffering capacity - Malocclusion prevalence - Caries prevalence and severity - Oral cleanliness - Periodontal condition - Prevalence of gingival bleeding - Gingival Index - Plaque prevalence	CINAHL, Ovid Embase, Ovid MEDLINE, PsycINFO, Web of Science	- Higher bruxism prevalence in ASD children - Lower salivary pH levels in ASD children - No significant differences in most variables assessed	Limited evidence, high risk of bias, need for further research.
Ningrum et al. [19]	- Decayed, missing, and filled permanent teeth (DMFT) index	PubMed, Scopus, Cochrane Library, Web of Science, Wiley Online Library	- Special needs children with intellectual disabilities or ASD had more caries - Positive correlation between caries prevalence and average DMFT value in their countries	Importance of an integrated and equitable care system to maintain oral health of special needs children, especially in high DMFT countries.

**Table 2:** Inferences obtained through an analysis of the selected reviews.

Source	Dental Caries	Periodontal Disease	Oral Hygiene
AlOtaibi et al. [14]	Not clearly specified	- Significantly higher gingival index values were found in children and adolescents with ASD than in children without ASD - -Pooled SMD used to infer no significant difference in the mean values of gingival index between the ASD and control groups (SMD = −0.528, t = 1.381, *p* = 0.168)	- Significantly higher plaque index values were found in children and adolescents with ASD than in children without ASD - The pooled SMD was used to infer no significant difference in the mean values of plaque indexes between the ASD and control groups (SMD = −0.687, t = 1.518, *p* = 0.129); individuals with ASD need help and better access to oral healthcare
Bartolomé et al. [15]	- Discrepancies found in studies regarding dental caries in children with ASD - No significant differences between children with ASD and control group - Greater incidence of dental caries among children with ASD - Significant differences with a greater predisposition to dental caries in non-autistic children	- Differences found in the evaluation of gingival statuses of children with ASD and control group - Higher gingival indexes for children with ASD - Statistically significant worse periodontal statuses in children with ASD - Statistically significant worse periodontal statuses in children with sensory impairment, primarily in those with visual deficits	- Significantly worse states of oral hygiene in children with ASD - One study found no differences, with oral hygiene being acceptable in both groups
Corridore et al. [16]	- No common DMFT or dmft for ASD group - Varied with age and compared to unaffected group	- Higher PI and GI for ASD group - Greater incidence of periodontal disease	- Poor oral hygiene - High prevalence of gingivitis and OHI-S
Da Silva et al. [17]	- Pooled prevalence of 60.6% (95% CI: 44.0–75.1) - Changed to 67.3% after sensitivity analysis	- Pooled prevalence of 69.4% (95% CI: 47.6–85.0) - Changed to 59.8% after sensitivity analysis	No statistical difference observed between the ASD and control groups
Lam et al. [18]	No statistical difference observed between the ASD and control groups	- Higher CPITN for ASD group than controls - OR = 5.72	- Poor oral hygiene - No significant difference in OHI-S (OR = 4.04) and plaque index (WMD = 0.31)
Ningrum et al. [19]	- Higher DMFT for special needs group than control group - SMD = 0.441	- Higher CPITN for special needs group - SMD = 1.419	- Higher OHI-S for special needs group than control group - SMD = 0.803; higher plaque index (SMD = 0.158); gingiva index with publication bias

## Data Availability

The data will be available from the corresponding authors and can be accessed upon request to the corresponding authors.

## References

[1] Ferrazzano G.F., Salerno C., Bravaccio C., Ingenito A., Sangianantoni G., Cantile T. (2020). Autism spectrum disorders and oral health status:review of the literature. Eur. J. Paediatr. Dent..

[2] El-Yousfi S., Jones K., White S., Marshman Z. (2019). A rapid review of barriers to oral healthcare for vulnerable people. Br. Dent. J..

[3] Rouleau T., Harrington A., Brennan M., Hammond F., Hirsch M., Nussbaum M., Bockenek W. (2011). Receipt of dental care and barriers encountered by persons with disabilities. Spéc. Care Dent..

[4] Lord C., Elsabbagh M., Baird G., Veenstra-Vanderweele J. (2018). Autism spectrum disorder. Lancet.

[5] Udhya J., Varadharaja M.M., Parthiban J., Srinivasan I. (2014). Autism disorder (AD): An updated review for paediatric dentists. J. Clin. Diagn. Res. JCDR.

[6] Alshatrat S.M., Al-Bakri I.A., Al-Omari W.M., Al Mortadi N.A. (2021). Oral health knowledge and dental behavior among individuals with autism in Jordan: A case–control study. BMC Oral Health.

[7] Gandhi R.P., Klein U. (2014). Autism Spectrum Disorders: An Update on Oral Health Management. J. Evid. Based Dent. Pract..

[8] Bernath B., Kanji Z. (2021). Exploring Barriers to Oral Health Care Experienced by Individuals Living with Autism Spectrum Disorder. Can. J. Dent. Hyg..

[9] Fontaine-Sylvestre C., Roy A., Rizkallah J., Dabbagh B., Ferraz dos Santos B. (2017). Prevalence of malocclusion in Canadian children with autism spectrum disorder. Am. J. Orthod. Dentofac. Orthop..

[10] Jaber M.A. (2011). Dental caries experience, oral health status and treatment needs of dental patients with autism. J. Appl. Oral Sci..

[11] Zhang Y., Lin L., Liu J., Shi L., Lu J. (2020). Dental Caries Status in Autistic Children: A Meta-analysis. J. Autism Dev. Disord..

[12] Page M.J., McKenzie J.E., Bossuyt P.M., Boutron I., Hoffmann T.C., Mulrow C.D., Shamseer L., Tetzlaff J.M., Akl E.A., Brennan S.E. (2021). The PRISMA 2020 statement: An updated guideline for reporting systematic reviews. BMJ.

[13] Aromataris E., Fernandez R., Godfrey C., Holly C., Khalil H., Tungpunkom P., Aromataris E., Munn Z. (2020). Chapter 10: Umbrella Reviews. JBI Manual for Evidence Synthesis.

[14] AlOtaibi A., Ben Shaber S., AlBatli A., AlGhamdi T., Murshid E. (2021). A systematic review of population-based gingival health studies among children and adolescents with autism spectrum disorder. Saudi Dent. J..

[15] Bartolomé-Villar B., Mourelle-Martínez M.R., Diéguez-Pérez M., de Nova-García M.J. (2016). Incidence of oral health in paediatric patients with disabilities: Sensory disorders and autism spectrum disorder. Systematic review II. J. Clin. Exp. Dent..

[16] Corridore D., Zumbo G., Corvino I., Guaragna M., Bossù M., Polimeni A., Vozza I. (2020). Prevalence of oral disease and treatment types proposed to children affected by Autistic Spectrum Disorder in Pediatric Dentistry: A Systematic Review. Clin. Ter..

[17] da Silva S.N., Gimenez T., Souza R.C., Mello-Moura A.C.V., Raggio D.P., Morimoto S., Lara J.S., Soares G.C., Tedesco T.K. (2017). Oral health status of children and young adults with autism spectrum disorders: Systematic review and meta-analysis. Int. J. Paediatr. Dent..

[18] Lam P.P., Du R., Peng S., McGrath C.P., Yiu C.K. (2020). Oral health status of children and adolescents with autism spectrum disorder: A systematic review of case-control studies and meta-analysis. Autism.

[19] Ningrum V., Bakar A., Shieh T.-M., Shih Y.-H. (2021). The Oral Health Inequities between Special Needs Children and Normal Children in Asia: A Systematic Review and Meta-Analysis. Healthcare.

[20] Tomazoni F., Zanatta F.B., Tuchtenhagen S., da Rosa G.N., Del Fabro J.P., Ardenghi T.M. (2014). Association of gingivitis with child oral health-related quality of life. J. Periodontol..

[21] Blomqvist M., Bejerot S., Dahllöf G. (2015). A cross-sectional study on oral health and dental care in intellectually able adults with autism spectrum disorder. BMC Oral Health.

[22] Stein L.I., Polido J.C., Mailloux Z., Coleman G.G., Cermak S.A. (2011). Oral care and sensory sensitivities in children with autism spectrum disorders. Spec. Care Dentist..

[23] Rogers S.J., Ozonoff S. (2005). Annotation: What do we know about sensory dysfunction in autism? A critical review of the empirical evidence. J. Child Psychol. Psychiatry.

[24] Ashburner J., Bennett L., Rodger S., Ziviani J. (2013). Understanding the sensory experiences of young people with autism spectrum disorder: A preliminary investigation. Aust. Occup. Ther. J..

[25] Subramaniam P., Gupta M. (2011). Oral health status of autistic children in India. J. Clin. Pediatr. Dent..

[26] Rai K., Hegde A.M., Jose N. (2012). Salivary antioxidants and oral health in children with autism. Arch. Oral Biol..

[27] Lu Y.Y., Wei I.H., Huang C.C. (2013). Dental health—A challenging problem for a patient with autism spectrum disorder. Gen. Hosp. Psychiatry.

[28] Tofani M., Galeoto G., Cazzetta D., Berardi A., Sansoni J., Valente D. (2019). Validation of the Pediatric Evaluation of Disability Inventory in an Italian Population with Autism Spectrum Disorder: A Cross-Sectional Study. Clin. Ter..

[29] Ferrara R., Ansermet F., Massoni F., Petrone L., Onofri E., Ricci P., Archer T., Ricci S. (2016). Autism Spectrum Disorder and intact executive functioning. Clin. Ter..

[30] Romagnoli G., Leone A., Romagnoli G., Sansoni J., Tofani M., De Santis R., Valente D., Galeoto G. (2019). Occupational Therapy’s efficacy in children with Asperger’s syndrome: A systematic review of randomized controlled trials. Clin. Ter..

[31] Troili G.M., Businaro R., Massoni F., Ricci L., Petrone L., Ricci P., Ricci S. (2013). Indagine su un gruppo di bambini autistici: Possibili fattori di rischio e considerazioni medico sociali [Investigation on a group of autistic children: Risk factors and medical social considerations]. Clin. Ter..

[32] Vozza I., Cavallè E., Corridore D., Ripari F., Spota A., Brugnoletti O., Guerra F. (2016). Preventive strategies in oral health for special needs patients. Ann. Stomatol..

[33] Barros A., Mascarenhas P., Botelho J., Machado V., Balixa G., Bandeira Lopes L. (2022). Autism Spectrum Disorders and Malocclusions: Systematic Review and Meta-Analyses. J. Clin. Med..

[34] da Motta T.P., Owens J., Abreu L.G., Debossan S.A.T., Vargas-Ferreira F., Vettore M.V. (2022). Malocclusion characteristics amongst individuals with autism spectrum disorder: A systematic review and meta-analysis. BMC Oral Health.

[35] Erwin J., Paisi M., Neill S., Burns L., Vassallo I., Nelder A., Facenfield J., Devalia U., Vassallo T., Witton R. (2022). Factors influencing oral health behaviours, access and delivery of dental care for autistic children and adolescents: A mixed-methods systematic review. Health Expect..

